# MHD Stagnation-Point Flow of a Carreau Fluid and Heat Transfer in the Presence of Convective Boundary Conditions

**DOI:** 10.1371/journal.pone.0157180

**Published:** 2016-06-20

**Authors:** Masood Khan, Hashim, Ali Saleh Alshomrani

**Affiliations:** 1 Department of Mathematics, Quaid-i-Azam University, Islamabad, 44000, Pakistan; 2 Department of Mathematics, Faculty of Science, King Abdul-Aziz University, Jeddah, 21589, Saudi Arabia; China University of Mining and Technology, CHINA

## Abstract

In the present investigation we analyze the impact of magnetic field on the stagnation-point flow of a generalized Newtonian Carreau fluid. The convective surface boundary conditions are considered to investigate the thermal boundary layer. The leading partial differential equations of the current problem are altered to a set of ordinary differential equations by picking local similarity transformations. The developed non-linear ordinary differential equations are then numerically integrated via Runge-Kutta Fehlberg method after changing into initial value problems. This investigation explores that the momentum and thermal boundary layers are significantly influenced by various pertinent parameters like the Hartmann number *M*, velocity shear ratio parameter *α*, Weissenberg number *We*, power law index *n*, Biot number *γ* and Prandtl number Pr. The analysis further reveals that the fluid velocity as well as the skin friction is raised by the velocity shear ratio parameter. Moreover, strong values of the Hartmann number correspond to thinning of the momentum boundary layer thickness while quite the opposite is true for the thermal boundary layer thickness. Additionally, it is seen that the numerical computations are in splendid consent with previously reported studies.

## Introduction

It is renowned fact that the magnetohydrodynamic (MHD), which is the science of motion of electrically conducting fluids, is one of the thrust areas of modern research. The elementary examples of electrically conducting fluids include plasmas, liquid metals (mercury or liquid sodium) and electrolytes. The basic theme of MHD is that if we place an electrically conducting fluid in magnetic field then motion of fluid may create a force called electromotive force. The electromotive force has the ability to induce current. Ever since then, this field has a broad spectrum of science and engineering, specifically in geophysics, fusion reactors, dispersion of metals, modern metallurgy and MHD generators *etc*. Moreover, MHD flows are of immense concern in problems related with physiological fluids. Pavlov [1] was the pioneer who discussed the influence of magnetic field on MHD flow past a stretching surface. Another eminent contribution was given by Andersson [2] who examined the MHD flow of a viscous fluid. Further, Fang and Zhang [3] reported the exact solution for hydrodynamic flow caused by a shrinking sheet with wall mass suction. The partial slip effects on hydromagnetic flow with heat source/sink and thermal radiation was also investigated by Hakeem *et al*. [4]. Recently, Makinde *et al*. [5] scrutinized the MHD variable viscosity reacting flow over a convectively heated plate in a porous medium with thermophoresis and radiative heat transfer. Moreover, Nadeem *et al*. [6] deliberated the influence of MHD and partial slip on an oblique stagnation-point flow of a rheological fluid by stretching surface. In this work, they found that both the tangential and normal velocities of fluid are depressed by the magnetic field.

For the time being a number of findings have been reported on the boundary layer flow of different fluids in the region of stagnation point. From both theoretical and experimental standpoints stagnation point flow has attracted many scientist and researchers due to its immense applications in real world and industrial processes. These processes include glass blowing, cooling and drying of papers, continuous casting of fibers, cable coating and other industrial processes in engineering. The fluid pressure, heat transfer and rate of mass deposition are highest in the area of stagnation-point. The study of a stagnation-point flow over a solid boundary in moving fluid was proposed by Hiemenz [7] in 1911. He was the inaugural who discussed the two-dimensional stagnation-point flow towards a stationary semi-infinite sheet. He reduced the Navier-Stokes equations into ordinary differential equations by practicing the similarity variables. Later on his idea was extended by various authors to study the different aspects of stagnation-point flow problems. Eckert [8] expended the Hiemenz work by considering the energy equation and calculated the exact solution corresponding to the thermal field. Further, stagnation-point flow over a stretching sheet was studied by Chiam [9]. In his work, he discussed the problem by taking the stretching velocity of the plate equal to straining velocity. Mahapatra and Gupta [10] reconsidered this problem by assuming different stretching and straining velocities and observed two different types of boundary layers.

In the recent years, the flow and heat transfer in non-Newtonian fluids have been studied by various authors. Many attempts are reported on non-Newtonian fluids such as power law and Sisko fluids. Postelnicu and Pop [11] studied the Falkner-Skan flow of a power-law fluid over a stretching wedge. Khan and Shahzad [12] discussed the flow behavior of Sisko fluid near the stagnation point towards a stretching surface. The flow and heat transfer to Sisko nanofluid over a stretching sheet was studied by Khan *et al*. [13]. However, amongst non-Newtonian fluids a little attention has been given to the Carreau model frequently used in chemical engineering. These types of fluids are known as generalized Newtonian fluids and they are discussed in detail in Bird *et al*. [14]. The Carreau fluid fits the suspensions of polymers behavior in several flow problems. It represents the pure viscous fluids in which the viscosity changes with the the deformation rate. Sobh [15] made the theoretical analysis of peristaltic motion of a non-Newtonian Carreau fluid in an asymmetric channel. Further, the analytic solutions are presented by Ali and Hayat [16] for the flow of generalized Newtonian Carreau fluid model with sinusoidal wall conditions. Martins *et al*. [17] discussed the numerical investigation of inertia and shear thinning effects in axisymmetric flows of Carreau fluids. The flow of the Carreau fluid around the spheres was discussed by Chhabra and Uhlherr [18]. Laterly, the flow and heat transfer to Carreau fluid in an annular space between two concentric cylinders was reported by Khellaf and Lauriat [19]. Recently, Khan and Hashim [20] studied the boundary layer flow to Carreau fluid over a non-linear stretching sheet.

The present study focuses on the numerical investigation of magnetohydrodynamic (MHD) flow of a non-Newtonian Carreau fluid near a stagnation point. We perform the analysis of energy transport in aspect of convective surface conditions. The boundary layer equations given as a set of partial differential equations are first changed into nonlinear ordinary differential equations ahead being solved numerically via shooting technique with the Runge-Kutta Fehlberg method. In this paper, the physical significance of the controlling parameters on the velocity and temperature profiles are analyzed and discussed graphically. It should be noticed that the results of this study are greatly affected by the Hartmann number, velocity ratio parameter, power law index and Biot number.

## Problem Formulation

### Flow analysis

**Fig 1:**
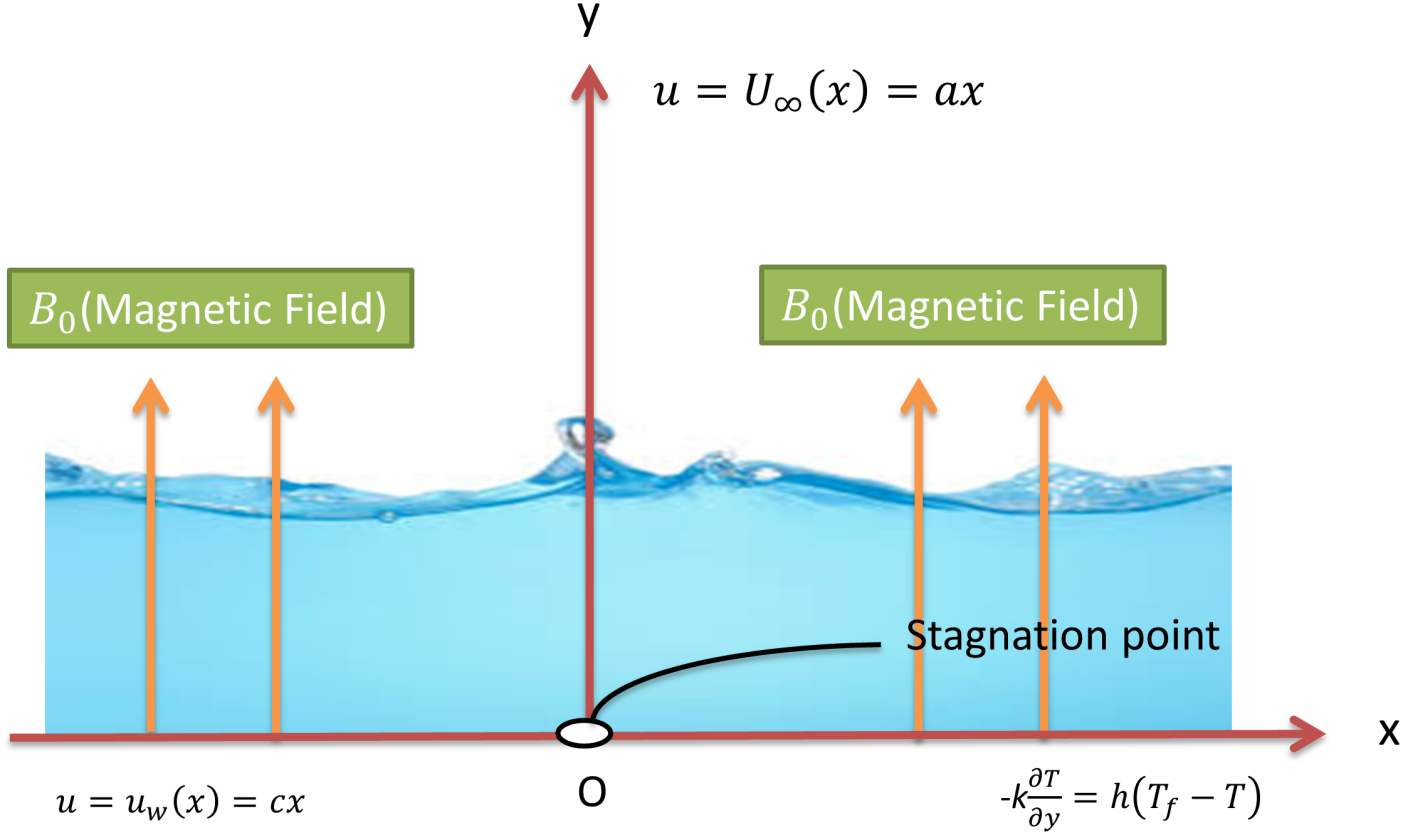
A schematic of the configuration of the physical model.

We consider a steady two-dimensional flow of an incompressible and electrically conducting Carreau fluid in the region of stagnation point towards a stretching surface. The Cartesian coordinate system (*x*,*y*) is chosen so that the *x*− axis is along the direction of sheet and *y*−axis is normal to it and the flow takes place at *y* > 0 as shown in Fig 1. The flow is originated by virtue of linear stretching of the sheet which is resulting from the employment of two equal and opposite forces. It is assumed that the magnetic field is of uniform strength *B*_0_ and applied transversely to the direction of the flow. The induced magnetic field is neglected under the small magnetic Reynolds number assumption. The sheet is stretching with the velocity *u_w_*(*x*) = *cx*, where *c* > 0 is the stretching rate. The velocity of external flow is *U*_∞_(*x*) = *ax*, where *a* > 0 represents a constant.

The essential equations for generalized Newtonian Carreau fluid can be written as [14]

$$\tau =-pI+\eta A_{1},$$ (1)

$$\eta =\eta_{\infty }+\left(\eta_{0}-\eta_{\infty }\right)\;{\left[1+{\left(\Gamma \overset{•}{\gamma }\right)}^{2}\right]}^{\frac{n-1}{2}},$$ (2)

where **τ** represents the Cauchy stress tensor, *p* the pressure, *n* the power law index, **I** the identity tensor, *η*_0_ the zero shear rate viscosity, *η*_∞_ the infinite shear rate viscosity, Γ a material parameter and **A**_1_ = (grad **V**)+(grad **V**)^T^ denotes first Rivlin-Ericksen tensor where **V** the velocity vector. The shear rate $\overset{•}{\gamma }$ for the flow is given by

$$\overset{•}{\gamma }=\sqrt{\frac{1}{2}\sum_{i}\sum_{j}{\overset{•}{\gamma }}_{ij}{\overset{•}{\gamma }}_{ji}}=\sqrt{\frac{1}{2}tr\left(A_{1}^{2}\right)}.$$ (3)

For many physical problems, we can take *η*_0_ >> *η*_∞_ (see [21]). The constitutive relation for *η*_∞_ = 0 is then

$$\eta =\eta_{0}{\left[1+{\left(\Gamma \overset{•}{\gamma }\right)}^{2}\right]}^{\frac{n-1}{2}}.$$ (4)

Under the above assumptions, the governing equations of conservation of mass and momentum in the presence of magnetic field can be written as [20]:

$$\frac{\partial u}{\partial x}+\frac{\partial v}{\partial y}=0,$$ (5)

u∂u∂x+v∂u∂y=U∞∂U∞∂x+η0ρ(∂2u∂x2+∂2u∂y2) [1+Γ2{4(∂u∂x)2+(∂u∂y+∂v∂x)2}]n−12+2η0ρ∂u∂x∂∂x[1+Γ2{4(∂u∂x)2+(∂u∂y+∂v∂x)2}]n−12+η0ρ(∂u∂y+∂v∂x) ∂∂y[1+Γ2{4(∂u∂x)2+(∂u∂y+∂v∂x)2}]n−12+σB02ρ(U∞−u), (6)

u∂v∂x+v∂v∂y=η0ρ(∂2v∂x2+∂2v∂y2) [1+Γ2{4(∂u∂x)2+(∂u∂y+∂v∂x)2}]n−12+2η0ρ∂v∂y∂∂y[1+Γ2{4(∂u∂x)2+(∂u∂y+∂v∂x)2}]n−12+η0ρ(∂u∂y+∂v∂x) ∂∂x[1+Γ2{4(∂u∂x)2+(∂u∂y+∂v∂x)2}]n−12, (7)

where *ρ*, *σ*, *u* and *v* are the fluid density, electrical conductivity and the velocity components in *x* and *y* directions, respectively.

After applying the usual boundary-layer analysis, the ruling equations of motion become:

$$\frac{\partial u}{\partial x}+\frac{\partial v}{\partial y}=0,$$ (8)

u∂u∂x+v∂u∂y=U∞∂U∞∂x+ν∂2u∂y2[1+Γ2(∂u∂y)2]n−12+ν(n−1)Γ2∂2u∂y2(∂u∂y)2[1+Γ2(∂u∂y)2]n−32+σB02ρ(U∞−u), (9)

in which $\nu =\frac{\eta_{0}}{\rho }$ represents the kinematic viscosity of fluid.

The following boundary conditions are imposed on relevant velocity components:

$$u=u_{w}=cx,\;v=0\;,\text{ at }y=0,$$ (10)

$$u\to U_{\infty }=ax,\text{ as }y\to \infty .$$ (11)

To get the non-dimensional problem, we employ the following local similarity transformations:

$$\psi (x,\;y)=x\sqrt{c\nu }f(\eta ),\;\eta =y\sqrt{\frac{c}{\nu }}.$$ (12)

We choose the stream function *ψ*(*x*,*y*) such that the velocity components are given as:

$$u=\frac{\partial \psi }{\partial y},\;v=-\frac{\partial \psi }{\partial x}.$$ (13)

Using the above mentioned non-dimensional variables, Eq 9 gives a third order non-linear ordinary differential equation of the form:

$$\left[1+nWe^{2}{\left(f^{\prime \prime }\right)}^{2}\right]\;{\left[1+We^{2}{\left(f^{\prime \prime }\right)}^{2}\right]}^{{}^{\frac{n-3}{2}}}f^{\prime \prime \prime }+ff^{\prime \prime }-{\left(f^{\prime }\right)}^{2}+\alpha^{2}+M^{2}(\alpha -f^{\prime })=0.$$ (14)

In above equation *α* = *a* / *c* is known as the velocity shear ratio parameter,$M=\sqrt{\frac{\sigma B_{0}^{2}}{\rho c}}$ the Hartmann number and $We=\sqrt{\frac{c^{3}\Gamma^{2}x^{2}}{\nu }}$ the local Weissenberg number.

In view of Eq 12, the associated boundary conditions can be written as:

$$f(0)=0,\;f^{\prime }(0)=1,\;f^{\prime }(\infty )\to \alpha .$$ (15)

The important physical quantity of practical interest in this problem is the local skin friction *C*_*f*_, which has the form

$$C_{f}=\frac{\tau_{w}}{\rho U_{w}^{2}(x)},$$ (16)

where the wall shear stress *τ*_*w*_ is given by

$$\tau_{w}={\eta_{0}\frac{\partial u}{\partial y}{\left[1+\Gamma^{2}{\left(\frac{\partial u}{\partial y}\right)}^{2}\right]}^{\frac{n-1}{2}}|}_{y=0}.$$ (17)

By using the above equation, an expression for the skin friction becomes

$$Re^{1/2}C_{f}=f^{\prime \prime }(0){\left[1+We^{2}{\left(f^{\prime \prime }(0)\right)}^{2}\right]}^{\frac{n-1}{2}}.$$ (18)

### Heat transfer analysis

Let us consider the energy transfer for the flow of Carreau fluid in the region of stagnation point towards a stretching surface. Using boundary layer approximations and neglecting viscous dissipation, the transfer of heat to the Carreau fluid flow is governed by the energy equation:

$$\rho c_{p}\left(u\frac{\partial T}{\partial x}+v\frac{\partial T}{\partial y}\right)=k\frac{\partial^{2}T}{\partial y^{2}}.$$ (19)

In Eq 19 *c*_*p*_, *k* and *T* are the specific heat, thermal conductivity and the fluid temperature, respectively. In order to apply the appropriate boundary conditions, the hot fluid with temperature *T*_*f*_ is used to heat up or cool down the surface of the sheet by taking convective heat transfer mode, which gives the convective heat transfer coefficient *h*. The boundary conditions are then:

$$k\frac{\partial T}{\partial y}=-h\left[T_{f}-T\right]\text{ at }y=0,$$ (20)

$$T\to T_{\infty }\text{ as }y\to \infty .$$ (21)

in which *T*_∞_ is the ambient fluid temperature.

The dimensionless temperature *θ* is defined by:

$$\theta =\frac{T-T_{\infty }}{T_{f}-T_{\infty }}.$$ (22)

In view of Eq 22 the energy equation Eq 19 and the boundary conditions 20 and 21 can be written as:

$$\theta^{\prime \prime }+\text{Pr}f\theta^{\prime }=0,$$ (23)

$$\theta^{\prime }(0)=-\gamma \left[1-\theta (0)\right],\;\theta (\infty )=0.$$ (24)

Here $\text{Pr}=\frac{\mu c_{p}}{k}$ is the Prandtl number and $\gamma =\frac{h}{k}\sqrt{\frac{\nu }{c}}$ represents the Biot number.

The expression for the local Nusselt number is:

$$Nu=\frac{xq_{w}}{k\left(T_{f}-T_{\infty }\right)},$$ (25)

where the surface heat flux *q*_*w*_ satisfies:

$$q_{w}={-k\left(\frac{\partial T}{\partial y}\right)|}_{y=0}.$$ (26)

Using Eq 22 the dimensionless local Nusselt number is expressed as

$$\text{R}{\text{e}}^{-1/2}Nu=-\theta^{\prime }(0).$$ (27)

### Solution methodology

In this study, the Runge-Kutta Fehlberg method has been employed to analyze the flow of the Carreau fluid for the partially coupled ordinary differential equations Eqs 14 and 23 with associated boundary conditions 15 and 24. In order to apply this technique, Eqs 14 and 23 which are third order in *f*(*η*) and second order in *θ*(*η*) have been converted to a system of five first order simultaneous equations in five unknowns. Let us define the new variables as:

$$f=f_{1},\;f^{\prime }=f_{2},\;f^{\prime \prime }=f_{3},\;f_{3}^{\prime }=f^{\prime \prime \prime },\;\theta =f_{4},\;\theta^{\prime }=f_{5},\;f_{5}^{\prime }=\theta^{\prime \prime }.$$ (28)

Employing the above new variables to Eqs 14 and 23 we obtain the following equivalent forms of the momentum and energy equations as

$$\left[1+nWe^{2}{\left(f_{3}\right)}^{2}\right]\;{\left[1+We^{2}{\left(f_{3}\right)}^{2}\right]}^{{}^{\frac{n-3}{2}}}f_{3}^{\prime }+f_{1}f_{3}-{\left(f_{2}\right)}^{2}+\alpha^{2}+M^{2}(\alpha -f_{2})=0,$$ (29)

$$f_{5}^{\prime }+\text{Pr}f_{1}f_{5}=0.$$ (30)

The partially coupled governing Eqs 29 and 30 can be converted into initial value problem in the manner

$$f_{1}^{\prime }=f_{2},\;f_{2}^{\prime }=f_{3},\;f_{3}^{\prime }=\frac{-f_{1}f_{3}+f_{2}^{2}-\alpha^{2}-M^{2}(\alpha -f_{2})}{\left(1+nWe^{2}f_{3}^{2}\right)\;{\left(1+We^{2}f_{3}^{2}\right)}^{\frac{n-3}{2}}},$$ (31)

$$f_{4}^{\prime }=f_{5},\;f_{5}^{\prime }=-\text{Pr}f_{1}f_{5}.$$ (32)

Here prime represents the derivatives with respect to *η* and the relevant boundary conditions 15 and 24 become

$$f_{1}=0,\;f_{2}=1,\;f_{5}=-\gamma \left(1-f_{4}\right)\text{ at }\eta =0$$ (33)

$$f_{2}\to \alpha ,\;f_{4}\to 0\text{ as }\eta \to \infty .$$ (34)

To solve Eqs 31 and 32 along with conditions 33 and 34 as initial value problem, one requires the value of *f*_3_(0), *i*.*e*.*f"*(0) and *f*_5_(0), *i*.*e*. *θ'*(0). However, no such values are granted at the boundary. The corresponding initial value problem is being solved numerically using a systematic guessing for *f"*(0) and *θ'*(0) (the missing initial value) via the shooting technique until the boundary conditions at infinity, *i*.*e*. *f"*(∞) and *θ'*(∞) are fulfilled. We use the step-size *h* = 0.1 to acquire the solutions and set the convergence criterion up to 10^−6^. The end conditions granted within Eqs 15 and 24 are exchanged with value equal to 12 of similarity variable *η*_max_ i.e.

$$\eta_{\text{max}}=12,\;f^{\prime }\left(12\right)=\alpha ,\;\theta \left(12\right)=0.$$ (35)

The selection of *η*_max_ = 12 assures that all the numerical solutions approaches the asymptotic values accurately.

## Results and Discussion

A comprehensive numerical computation is conducted for various physical parameters involved in current problem. To determine the accuracy in favor of present numerical results, a comparison of obtained results for the skin friction and local Nusselt number are presented in Tables 1 and 2 for particular values of the velocity shear ratio parameter *α* when *M = 0*, *n* = 1 and *We* = 0. Table 1 demonstrates that the numerical values of the skin friction coefficient for distinct values of *α* are in superb accord with the results published in [22–24]. To further ratify our method, comparison of the local Nusselt number −*θ'*(0)for various values of the velocity ratio parameter *α* and Prandtl number Pr has been shown in Table 2. This comparison also shows a very good agreement with the results in the open literature.

**Table 1:** A comparison of values of *f*"(0) with previous results when *n* = 1.0, *We* = 0.0 and *M* = 0.0 for different values of *α*.

*α*	Mahapatra and Gupta [22]	Nazar *et al*. [23]	Ishak *et al*. [24]	Present study
0.01	_	_	-0.9980	-0.998028
0.1	-0.9694	-0.9694	-0.9694	-0.969387
0.2	-0.9181	-0.9181	-0.9181	-0.918107
0.5	-0.6673	-0.6673	-0.6673	-0.667262
2.0	2.0175	2.0176	2.0175	2.017487
3.0	4.7293	4.7296	4.7294	4.729260

**Table 2:** A comparison of the values of local Nusselt number −*θ'*(0) with *n* = 1.0, *We* = 0.0 for different values of Pr.

Pr	*α*	Mahapatra and Gupta [22]	Hayat [25]	Present study
1				
	0.1	0.603	0.602156	0.602157
	0.2	0.625	0.624467	0.624471
	0.5	0.692	0.692460	0.692451
1.5	0.1	0.777	0.776802	0.776807
	0.2	0.797	0.797122	0.797129
	0.3	0.863	0.864771	0.864806

**Fig 2:**
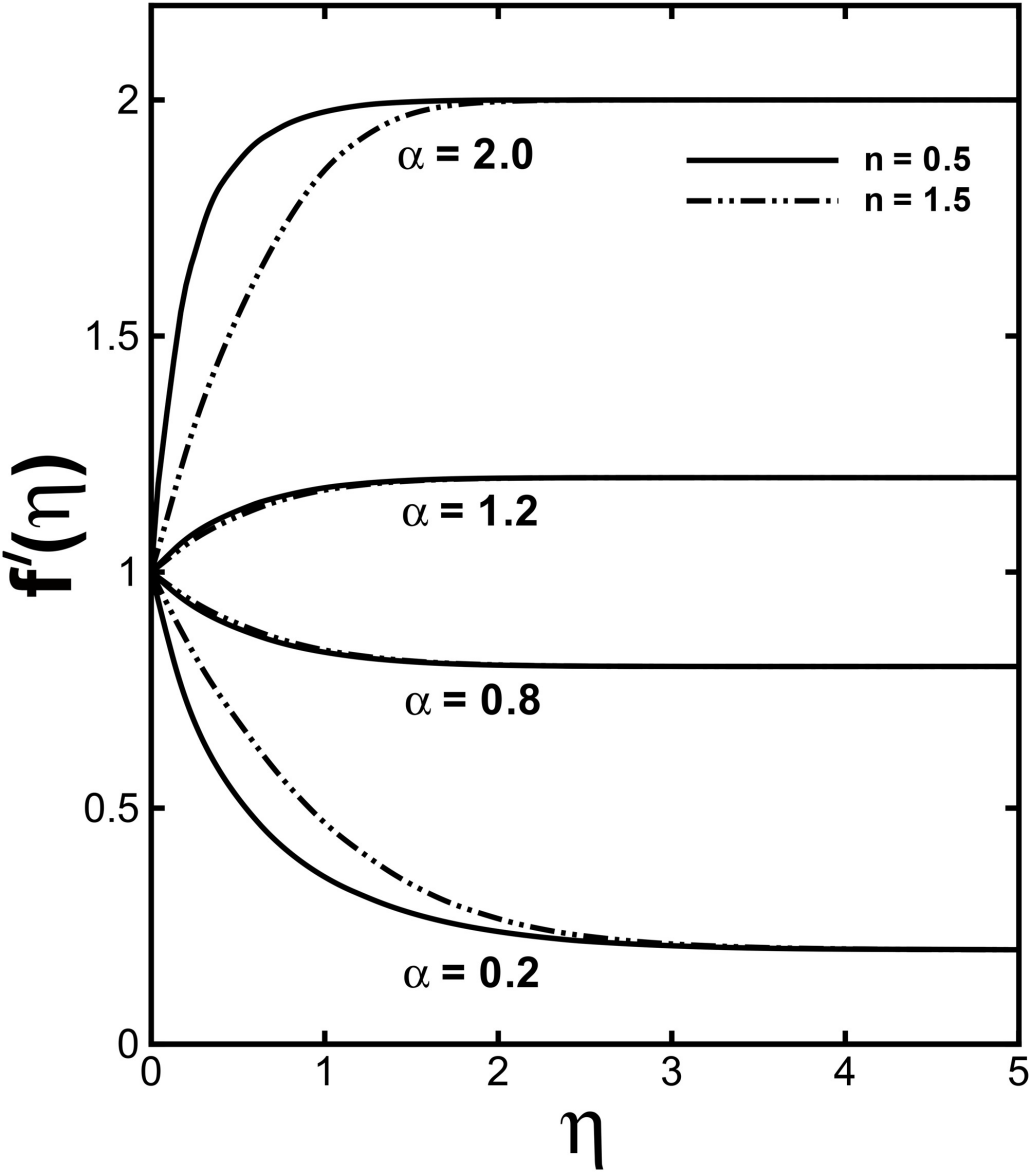
Effect of *α* on dimensionless velocity *f'*(*η*) when *M* = 0.5 and *We* = 3.0 are fixed.

**Fig 3:**
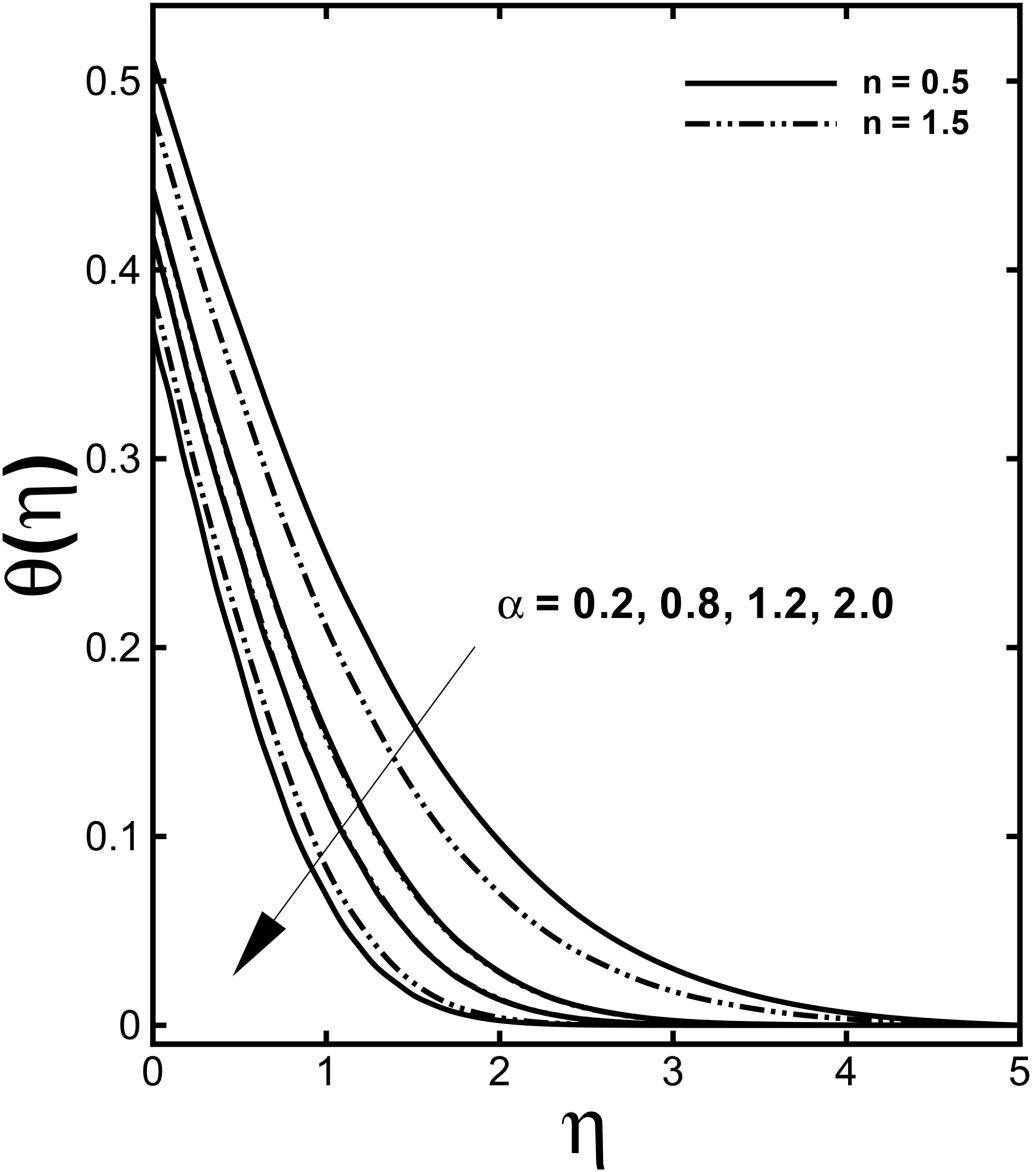
Effect of *α* on dimensionless temperature *θ*(*η*) when *M* = 0.5, *We* = 3.0, *γ =* 0.6 and Pr = 1.0 are fixed.

The profile of non-dimensional velocity *f'*(*η*) for varying values of the velocity shear ratio parameter *α* is visualized in Fig 2. It is analyzed that the velocity profile increases for higher values of *α* in both cases (*α* > 1 and *α* < 1) while the momentum boundary layer thickness predicts opposite behavior in both cases. This is due to the fact that *α* signifies the ratio of free stream velocity to the stretching velocity. We noticed that an increase in *α* means that the free stream velocity is greater than the sheet stretching velocity. According to Mahapatra and Gupta [10] as long as the fixed value of *c* accords to the stretching of the surface and an increment in *a* in relation to *c* assures a rise in the straining motion near the stagnation region that can enhances the acceleration of the external stream. Nevertheless, in case when plate velocity is higher than free stream velocity (*α* < 1), the flow shows an inverted boundary layer structure as a result the boundary layer thickness increases with a rise in *α* for (*α* < 1). Further, the boundary layer thickness is lesser for the shear thinning fluid (*n* < 1). The influences of the velocity shear ratio parameter *α* on temperature profiles are displayed in Fig 3. Temperature profiles depict a significantly decreasing trend with the higher values of *α*. Additionally, the thickness of the thermal boundary layer reduces for stronger *α*.

**Fig 4:**
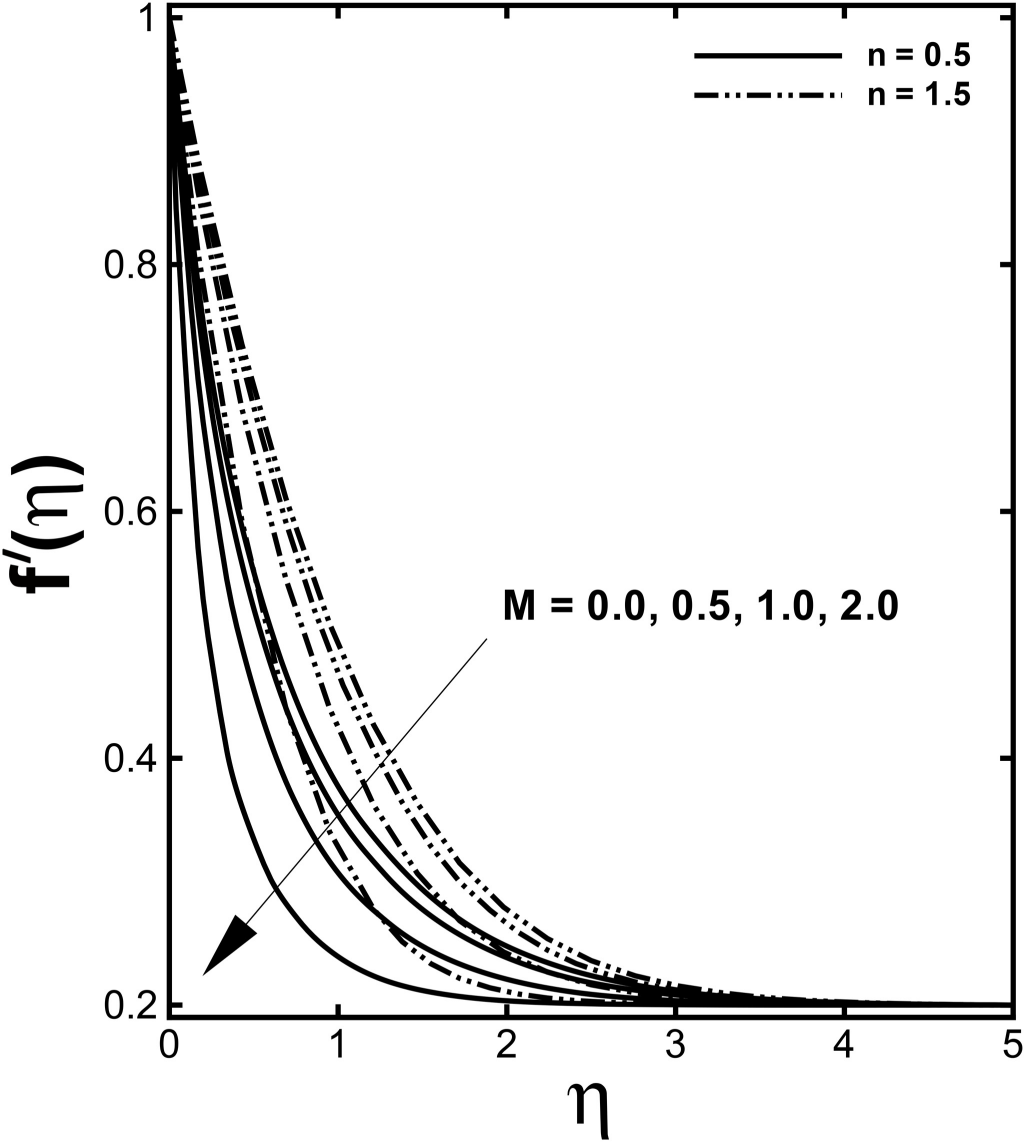
Effect of *M* on dimensionless velocity *f'*(*η*) when *α* = 0.2 and *We* = 3.0 are fixed.

**Fig 5:**
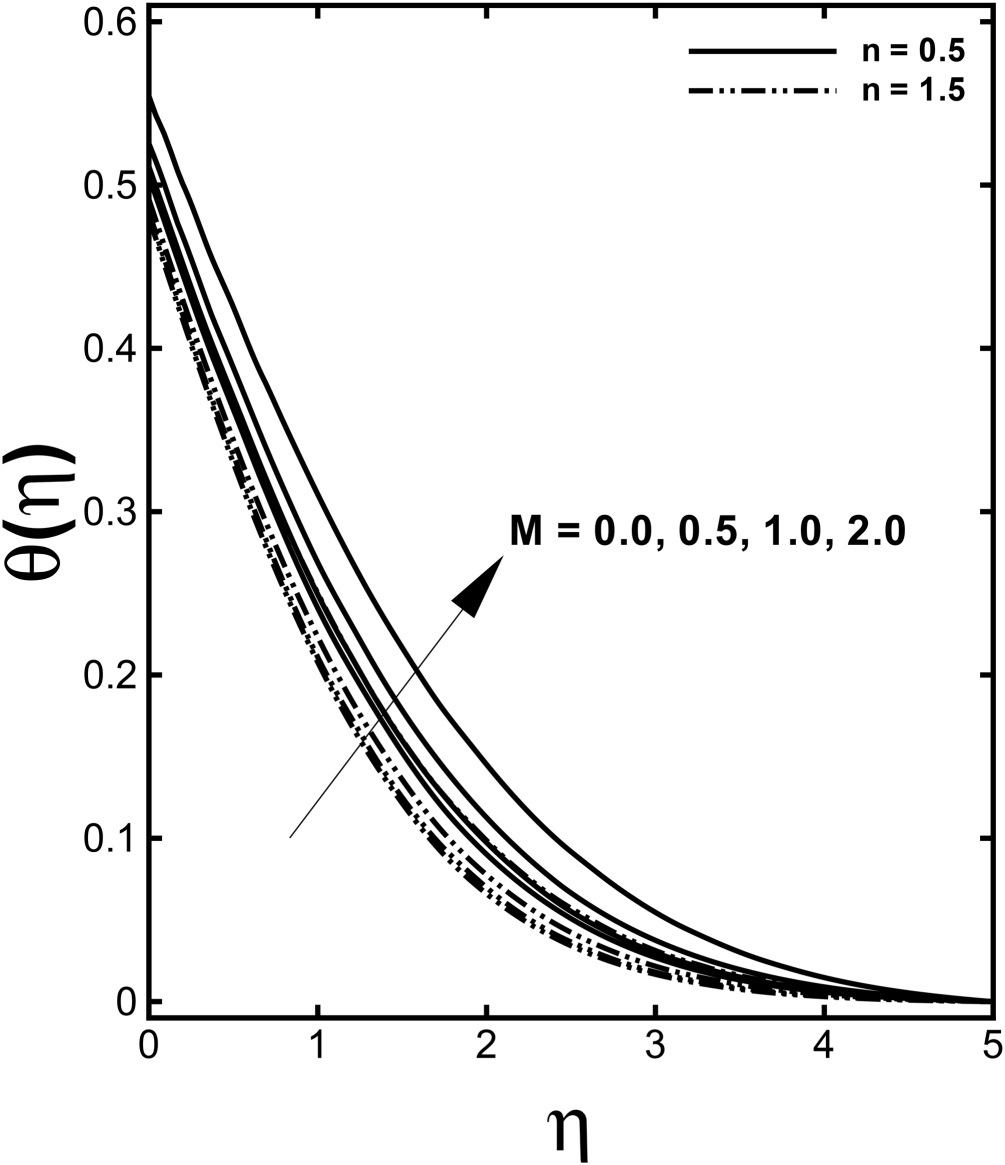
Effect of *M* on dimensionless temperature *θ*(*η*) when *α* = 0.2, *We* = 3.0, *γ =* 0.6 and Pr = 1.0 are fixed.

Figs 4 and 5 present the dimensionless velocity and temperature distributions for several values of the Hartmann number *M* for both the shear thinning and shear thickening fluids. We observed from these figures that a diminution in the velocity field and enhancement in the temperature field occur for increasing values of the Hartmann number *M*. This confirms the general physical behavior of the magnetic field that say that the fluid velocity depreciate for improved values of *M*. According to the physical point, *M* represents the ratio of electromagnetic force to the viscous force so large *M* implies that the Lorentz force increases, which is drag-like force that produces more resistance to transport phenomena due to which fluid velocity reduces. Consequently, the boundary layer thickness is a decreasing function of *M*. It is seen through Fig 5 that the temperature profile *θ*(*η*) enhances by uplifting the Hartmann number *M*. Practically, the Lorentz force has a resistive nature which opposes motion of the fluid and as a result heat is produced which increases thermal boundary layer thickness and fluid temperature.

**Fig 6:**
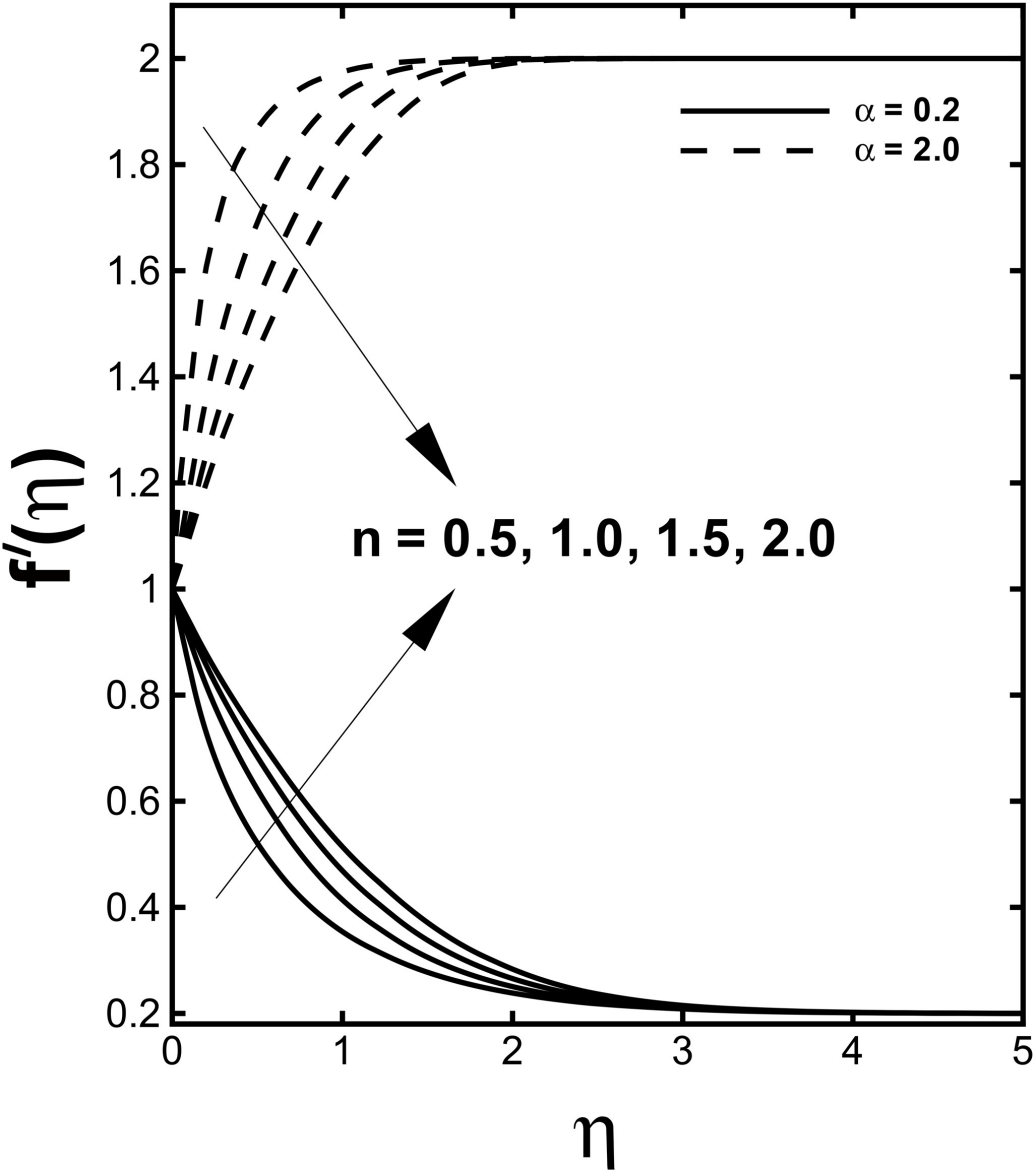
Effect of *n* on dimensionless velocity *f'*(*η*) when *M* = 0.5 and *We* = 3.0 are fixed.

**Fig 7:**
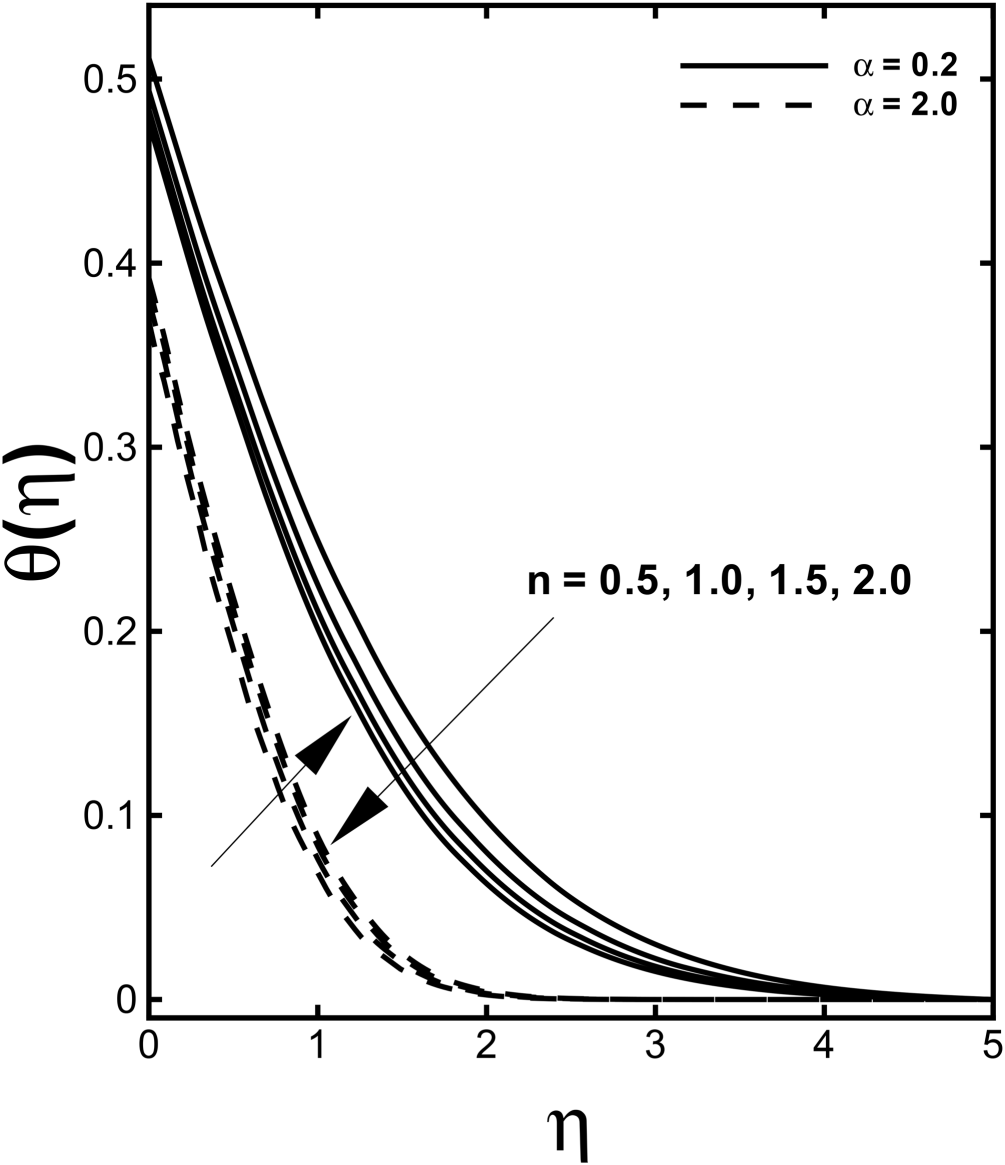
Effect of *n* on dimensionless temperature *θ*(*η*) when *M* = 0.5, *We* = 3.0, *γ =* 0.6 and Pr = 1.0 are fixed.

The variations of the velocity and temperature distributions for peculiar values of the power-law index *n* are illustrated through Figs 6 and 7. An increment in the power-law index *n* is responsible for the expansion in fluid velocity for the case when (*α* < 1) while an opposite outcome is seen for the case (*α* > 1) in Fig 6. The temperature profiles present shrinkage with the accelerating values of the power-law index *n* from 0.5 to 2 when the stretching velocity exceeds the free stream velocity. Moreover, when the stretching velocity is lower than the free stream velocity (*α* < 1) inflation in the temperature *θ*(*η*) is examined from Fig 7. The momentum boundary layer thickness arises while the thermal boundary layer thickness declines by increase of *n*.

**Fig 8:**
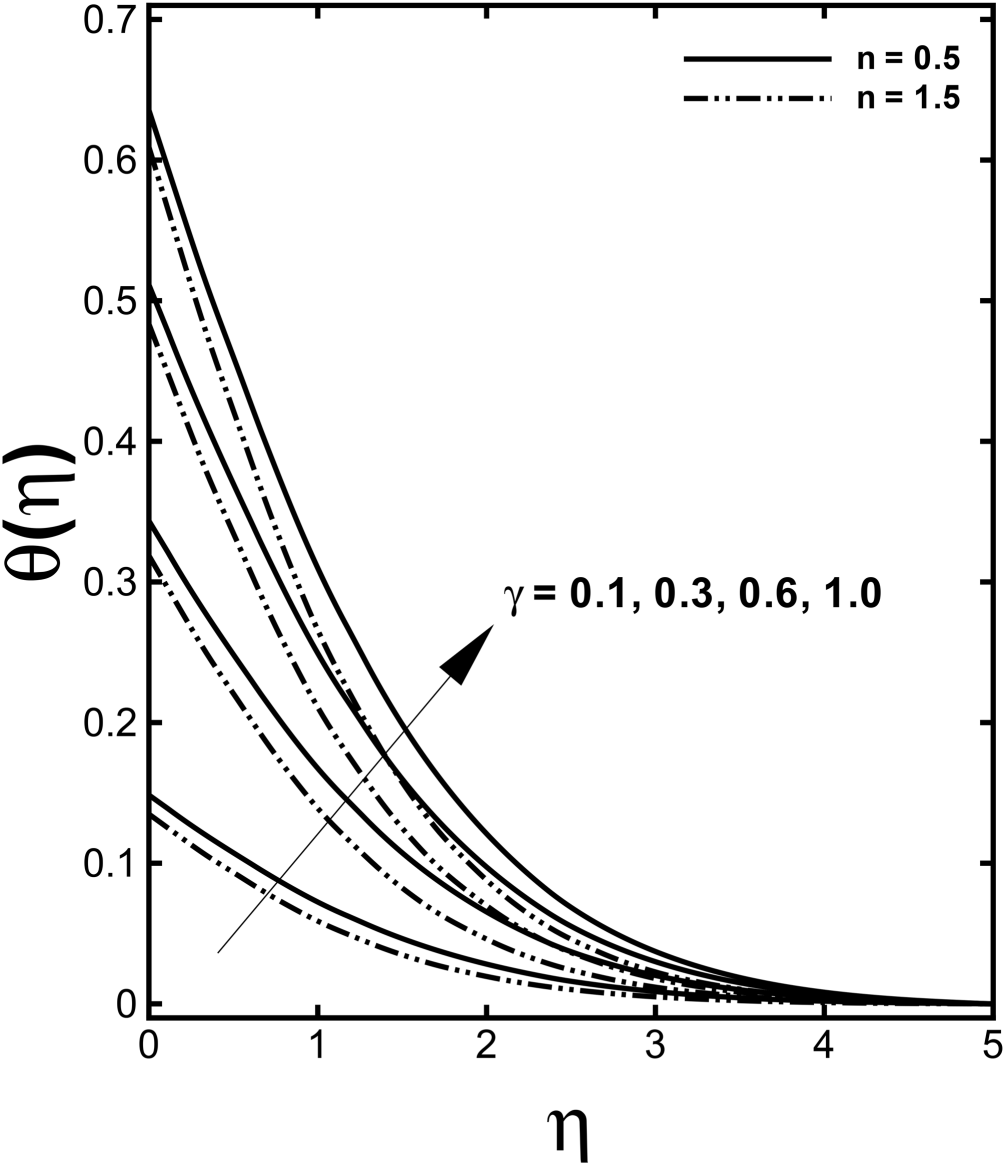
Effect of *γ* on dimensionless temperature *θ*(*η*) when *α* = 0.2, *M* = 0.5, *We* = 3.0 and Pr = 1.0 are fixed.

**Fig 9:**
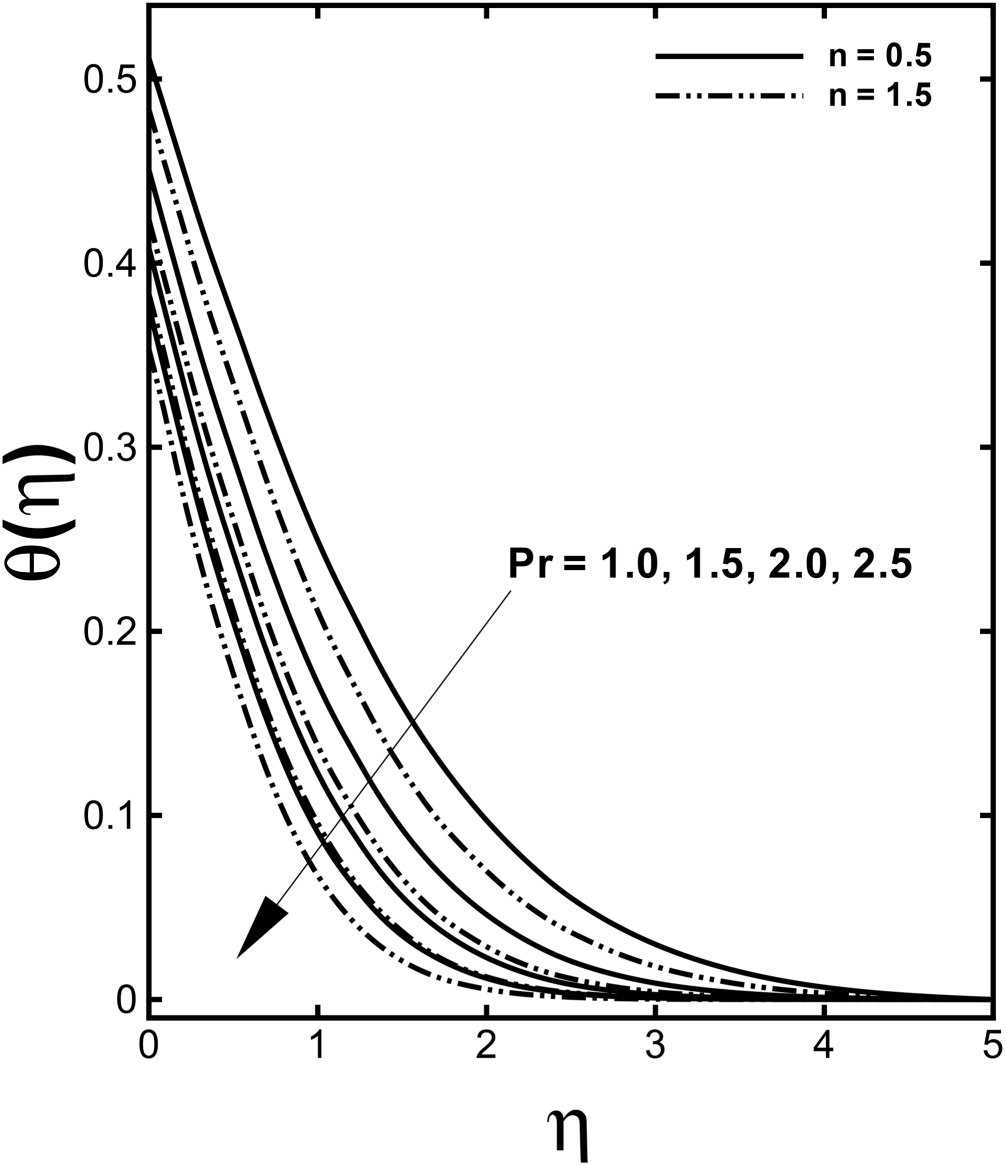
Effect of Pr on dimensionless temperature *θ*(*η*) when *α* = 0.2, *M* = 0.5, *We* = 3.0 and *γ =* 0.6 are fixed.

The impacts of the convective heating, known as Biot number *γ*, on the thermal field are depicted in Fig 8. The results are presented for different values of the power-law index *n* to compare the corresponding temperature profiles in case of shear-thinning (*n* < 1) and shear-thickening (*n* > 1) fluids. It is seen through this figure that increasing Biot number *γ* is responsible for higher fluid temperature. We know that physically Biot number implies the ratio of the convection at the surface to conduction within the surface of a body. Thus Biot number provides a way to compare the conduction resistance within a solid body to the convection resistance external to that body for heat transfer. Additionally, an increase in the Biot number means an increment in the convection at the surface due to which the surface temperature rises and thickness of the thermal boundary layer is significantly boosted. Fig 9 shows the effects of Prandtl number Pr on the heat transfer process. It is observed that the temperature of the fluid is depressed by larger Prandtl number. From a physical point of view, Prandtl number is a dimensionless number approximating the ratio of the momentum diffusivity to the thermal diffusivity. In short, a larger Pr feature lower thermal diffusion compared to viscous diffusion and hence it offers less penetration depth for temperature. Further, a decrease in the thermal boundary layer with strong Prandtl number is compensated with steeper temperature profiles.

**Fig 10:**
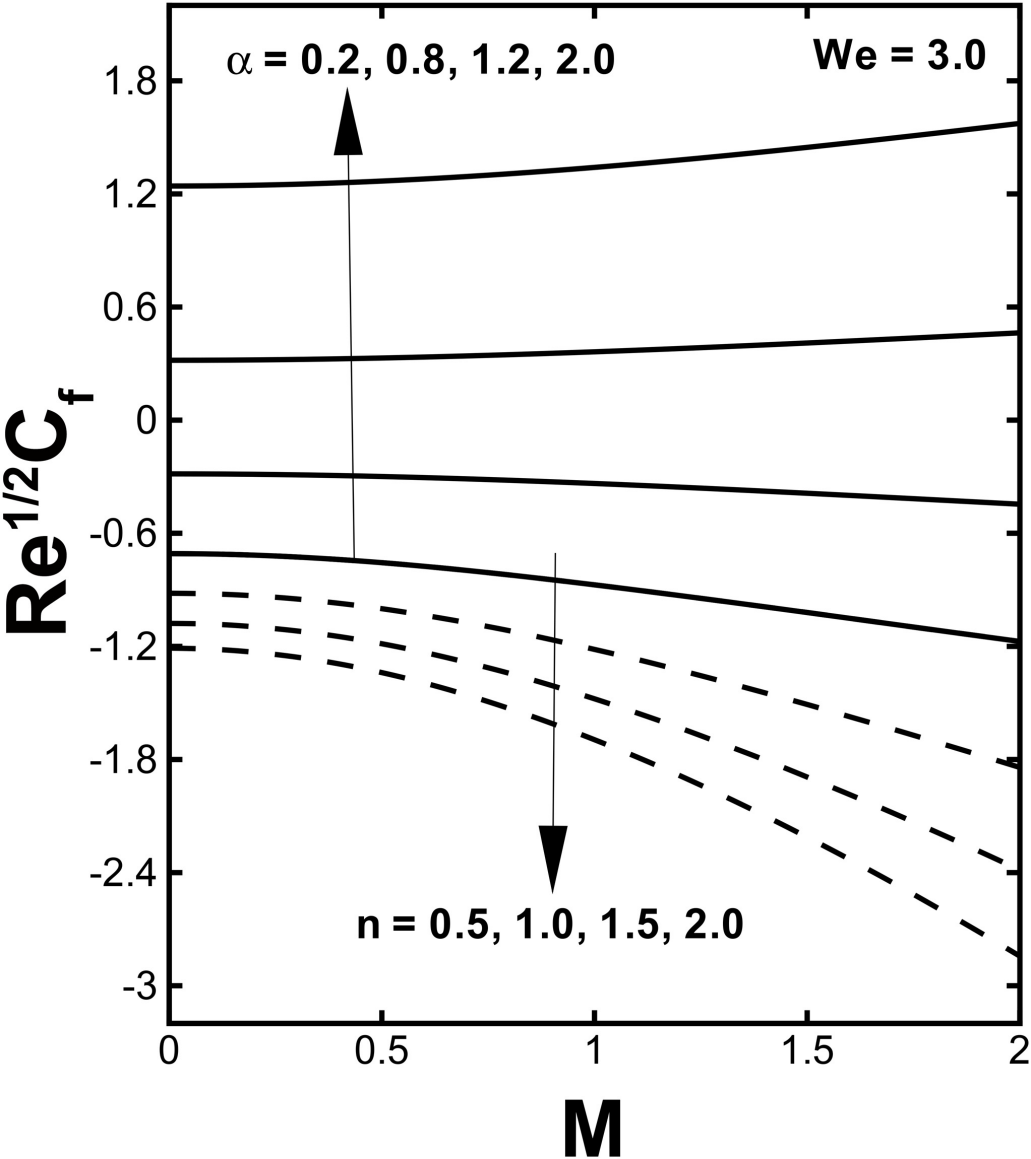
Variation of the local skin friction coefficient *C*_*f*_ against *M* for different values of *α* and *n*.

**Fig 11:**
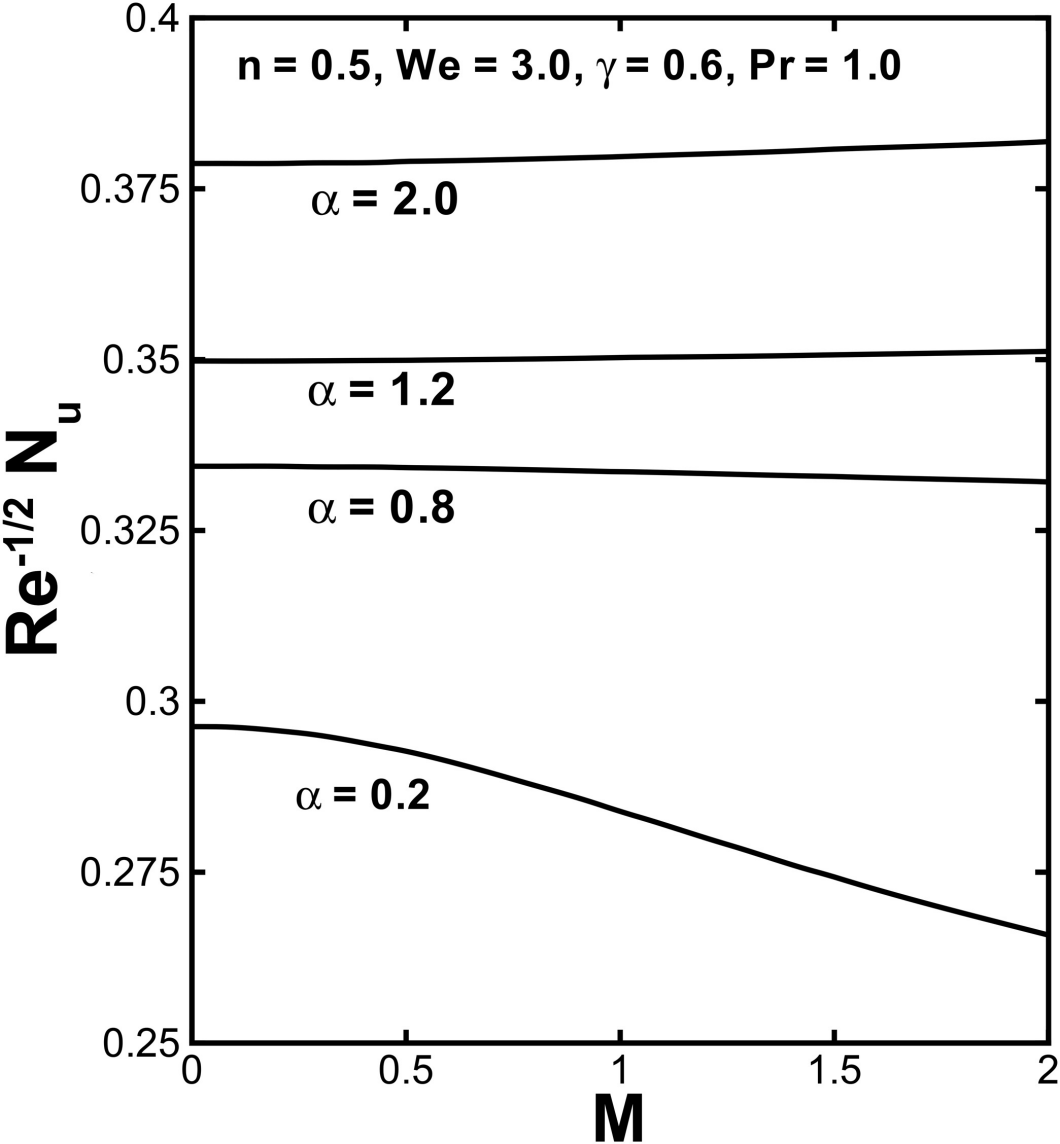
Variation of the local Nusselt number −*θ'*(0) against *M* for different values of *α*.

**Fig 12:**
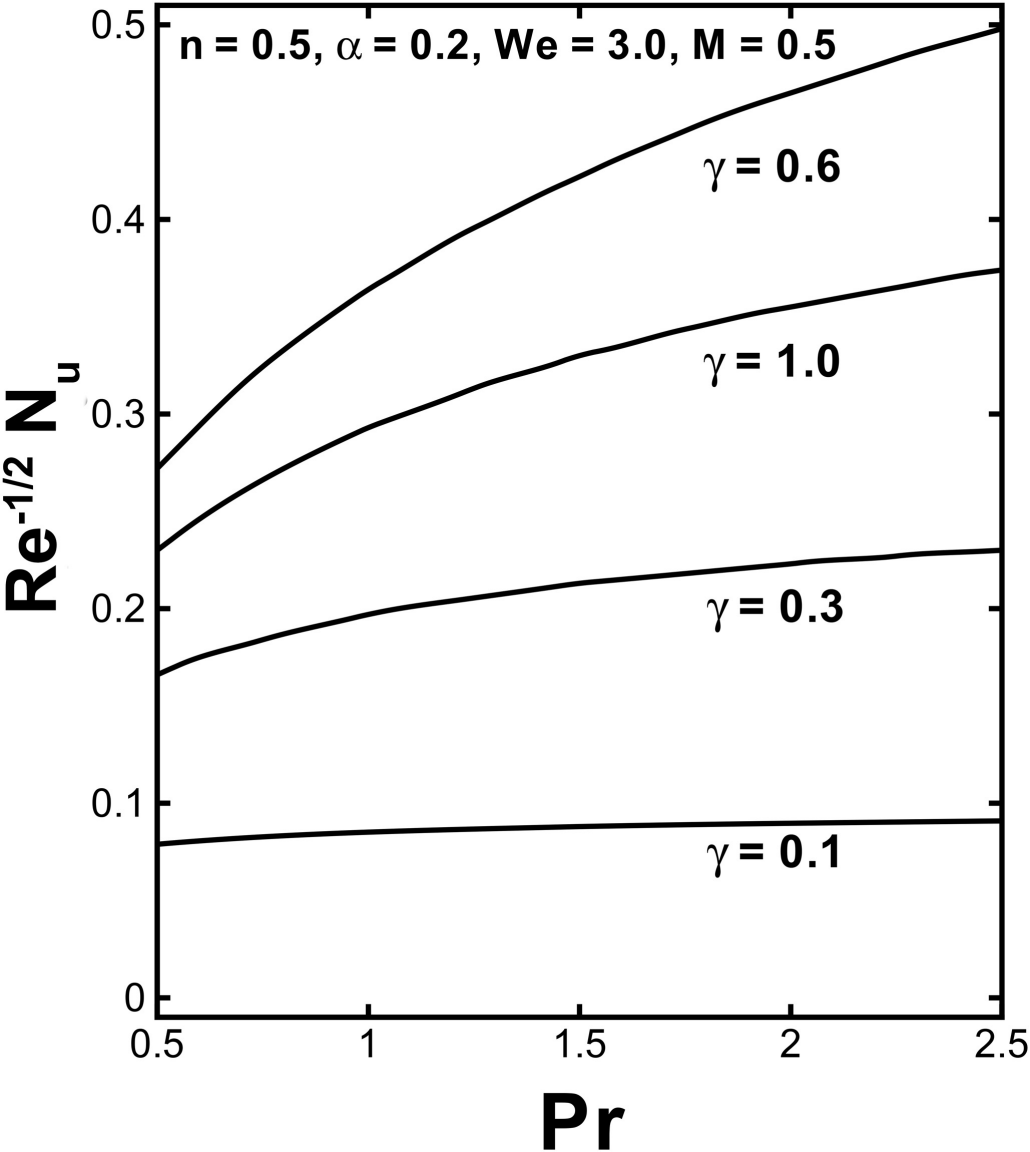
Variation of the local Nusselt number −*θ'*(0) against Pr for different values of *γ*.

The physical quantities, the skin friction *C*_*f*_ and the local Nusselt number *Nu*, which have massive engineering applications, are interpreted in Figs 10–12. The behavior of the skin friction against the Hartmann number *M* for various values of the velocity shear ratio parameter *α* and power law index *n* are plotted in Fig 10. This figure put in evidence that the wall skin friction rises with uplifting values of *α*. By increasing the Hartmann number *M* the skin friction decreases for (*α* < 1). While it increases with larger values of *M* in case of (*α* > 1). Fig 10 identifies that the magnitude of skin friction coefficient rises by enlarging the power law index *n*. The variation of the heat transfer rate at the wall −*θ'*(0) verses the Hartmann number *M* and Prandtl number Pr for distinct values of *α* and *γ* is shown in Figs 11 and 12. It can be concluded from Fig 11 that the rate of heat flux increases with the velocity shear ratio parameter. Likewise, a deterioration of the Nusselt number can be seen with higher magnetic parameter for (*α* > 1) Also an expansion in values of the local Nusselt number is expected for large *M* in case (*α* < 1). The local Nusselt number is proportional to slope of the temperature at *n* = 0and this rises with an enhancement in the Biot number.

## Conclusions

This article explored the heat transfer characteristics in magnetohydrodynamic flow of a non-Newtonian Carreau fluid towards a stretching surface. We further considered the convective boundary condition in this study. The governing partial differential equations of motion were reduced to a system of ordinary differential equations with the aid of local similarity variables. These ordinary differential equations were further solved by using the Runge-Kutta Fehlberg integration scheme. We gathered some important features regarding the different physical parameters of the problem. The following conclusions are drawn from current study:

1. The analysis exposed that an increase in the Hartmann number endorsed an expansion in the fluid temperature while a reverse behavior was perceived for the fluid velocity.
2. It was seen that the velocity profile declined inside the boundary layer in case when the stretching velocity dominates the free stream velocity (*α* < 1) while an opposite was true when free stream velocity dominates the stretching velocity (*α* > 1).
3. We found that the power law index has quite opposite effect on the velocity field for two different cases of velocity shear ratio parameter.
4. Influence of strong Biot number was to raise the thermal boundary layer thickness.
5. The increasing value of the velocity shear ratio parameter corresponded to an expansion in the rate of heat flux.
6. Magnitude of the skin friction was declined by uplifting the velocity shear ratio parameter when *α* < 1, whereas for *α* > 1 it was increased.
